# Prosthodontic Rehabilitation of a Completely Edentulous Patient With a Cleft Palate: A Case Report

**DOI:** 10.7759/cureus.33522

**Published:** 2023-01-08

**Authors:** Shreya Colvenkar, Rathod Prakash, Summiya Fatima, MD Shakeel Ahmed, Gayam Kishore Reddy

**Affiliations:** 1 Department of Prosthodontics, MNR Dental College & Hospital, Sangareddy, IND; 2 Department of Oral and Maxillofacial Surgery, MNR Dental College & Hospital, Sangareddy, IND

**Keywords:** congenital, prosthodontic rehabilitation, obturator, cleft palate, denture, edentulous

## Abstract

Clefts of the lip and palate are congenital anomalies that affect the orofacial region. Completely edentulous patients with a cleft palate present a unique challenge during complete denture fabrication. Complete dentures with an obturator and palatal extension need to be accurately fabricated to fulfill the functions of esthetics, speech, deglutition, and mastication. The case report presents the successful prosthodontic rehabilitation of a cleft palate female patient using a complete denture with a palatal obturator and pharyngeal extension. The palatal obturator extension provided good retention and stability for the maxillary denture. The patient’s skill and previous experience with her old dentures played an important role in successful treatment. The patient was satisfied with dentures in terms of esthetics, chewing, swallowing, and speech.

## Introduction

Clefts of the lip and palate are congenital anomalies that affect the orofacial region [1]. A literature search mentions that one-fourth of clefts are bilateral and three-fourths are unilateral. In the unilateral cleft palate, the left side is more involved than the right side.

The exact cause of the cleft lip/palate is unknown, and in most cases, no single factor can be identified. But various factors like smoking, medications, alcohol consumption, viruses, hypoxia, and nutritional deficiencies during pregnancy can produce clefts in the orofacial region [2].

The problems faced by cleft lip/palate patients are unique, and rehabilitation must address aesthetics, mastication, speech, and deglutition. The rehabilitation should be a team approach, comprising an oral surgeon, a prosthodontist, an orthodontist, a psychiatrist, and a speech therapist. The treatment starts from birth and continues for many years in some cases [2,3]. The treatment should include surgical closure as early as possible to allow patients to lead normal life.

When surgical closure is not done, treatment should concentrate on creating a dentition that allows optimal function, speech, and aesthetics. Patients with palatal defects and complete edentulism present a unique challenge during the fabrication of complete dentures. So proper obturator and palatal extension of the complete dentures need to be carried out to fulfill functions of speech, deglutition, and mastication [4].

This case report presents the successful rehabilitation of a cleft palate patient with a complete denture.

## Case presentation

**Figure 1:**
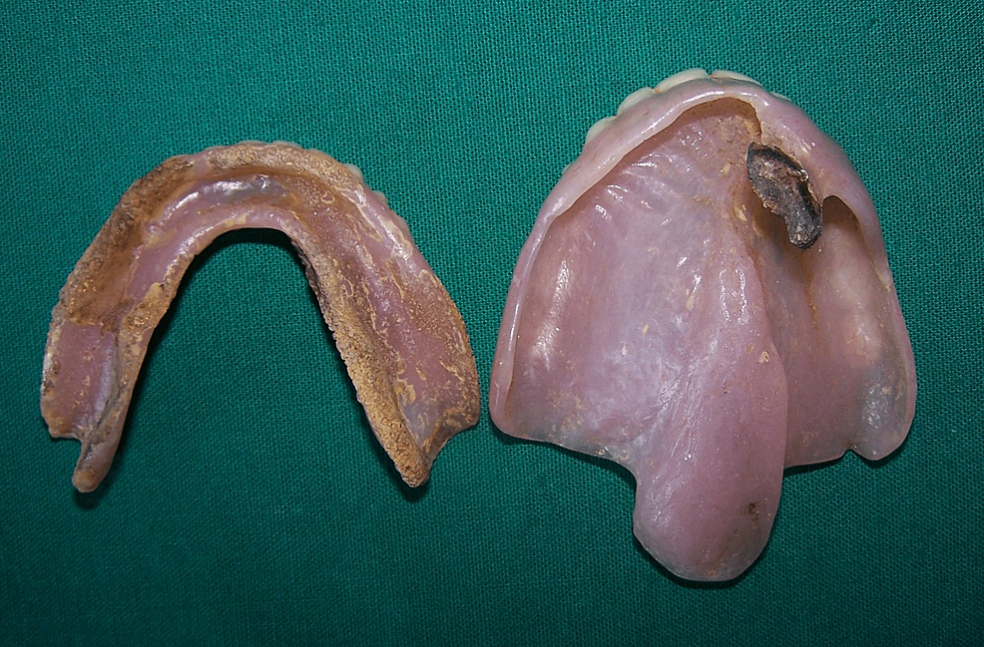
The patient's old dentures.

A 65-year-old female reported to the Department of Prosthodontics for the fabrication of new dentures. According to the history, the patient was born with a congenital cleft lip and palate. The cleft lip was repaired during the early years of her life, but the cleft palate was not surgically repaired. The patient was an old denture wearer for seven years, and recently, because of poor retention, she could not use her dentures for speaking, swallowing, or chewing (Figure 1).

**Figure 2:**
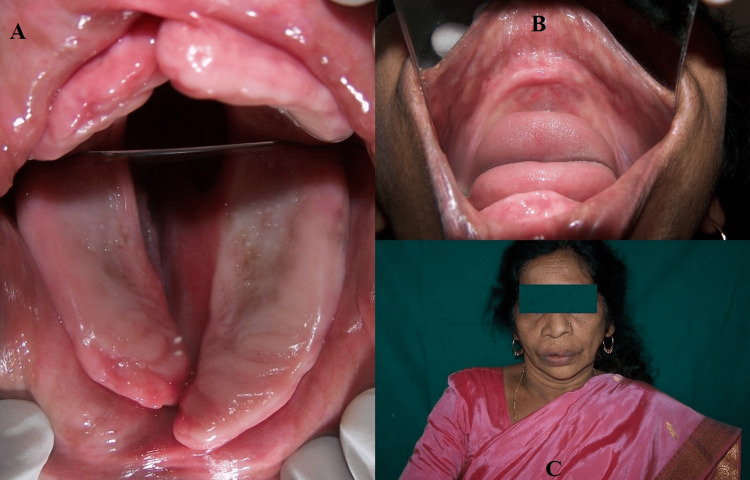
(A) Cleft palate. (B) Edentulous mandibular ridge. (C) Pretreatment extraoral view.

The patient’s straightforward request was to keep the design as similar to her old dentures as possible. On intraoral examination, a complete unilateral cleft was present involving the anterior alveolar bone, hard palate, and soft palate to the uvula (Figure 2).

**Figure 3:**
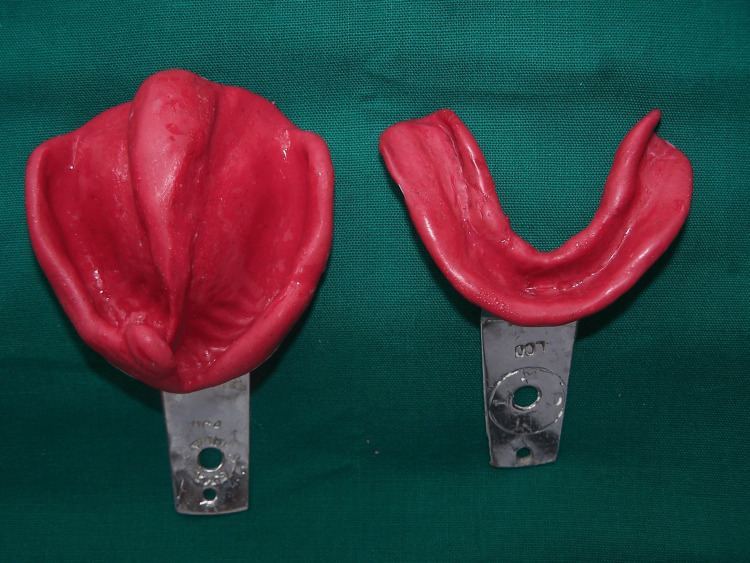
Primary impressions.

After selecting the appropriate stock impression tray, primary impressions were made with an impression compound (Figure 3). The undercuts on the primary cast were blocked with modeling wax in the cleft area.

Custom impression trays were fabricated with auto-polymerized acrylic resin. Border molding was carried out with a green stick impression compound. In the defect area, the type I modeling plastic impression compound was softened and adapted. To record the posterior extension of the denture, the patient was asked to bend the head forward slowly, touch the chest, and then move it backward. To record the lateral side, a softened modeling plastic impression compound was added and the patient was asked to do a side-to-side movement. The patient was also asked to say *ah* phonate repeatedly and forcefully. The impression surface was adjusted till the patient was satisfied with the speech and comfort. Secondary impressions were made with light-body elastomeric impression material.

**Figure 4:**
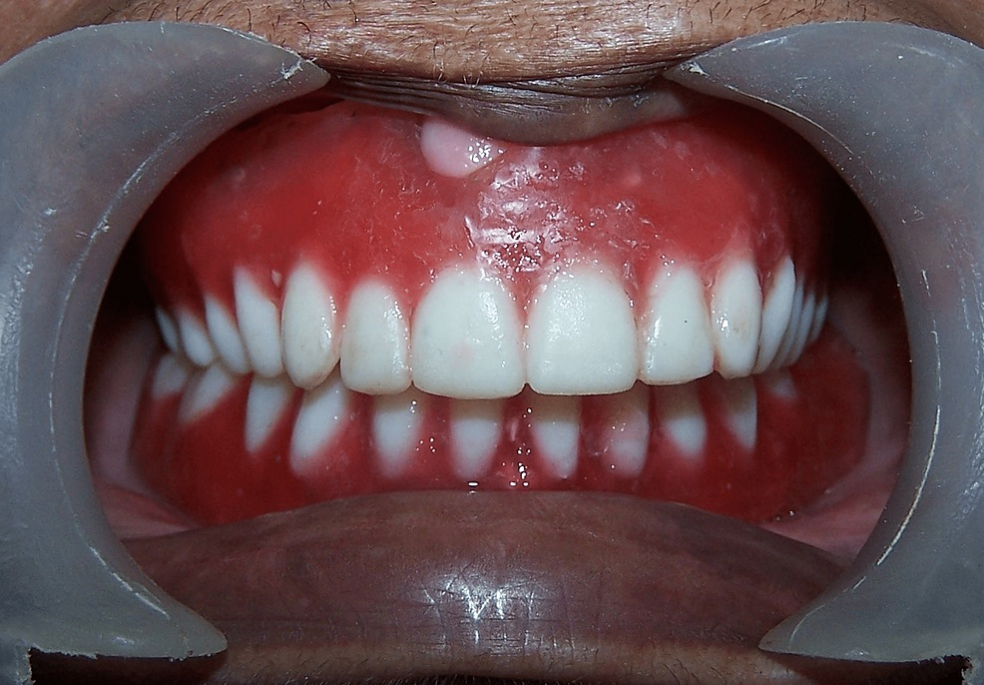
Try-on.

Jaw relations were recorded, followed by a try-on (Figure 4). During try-on, speech, aesthetics, and swallowing were evaluated. The patient's speech was evaluated using speech tests like perceptual analyses, resonance frequency analyses, and acoustic analyses. There was a marked improvement in speech. The patient was asked to drink water to check for regurgitation of liquid during swallowing. The denture prevented the regurgitation of liquid during swallowing.

**Figure 5:**
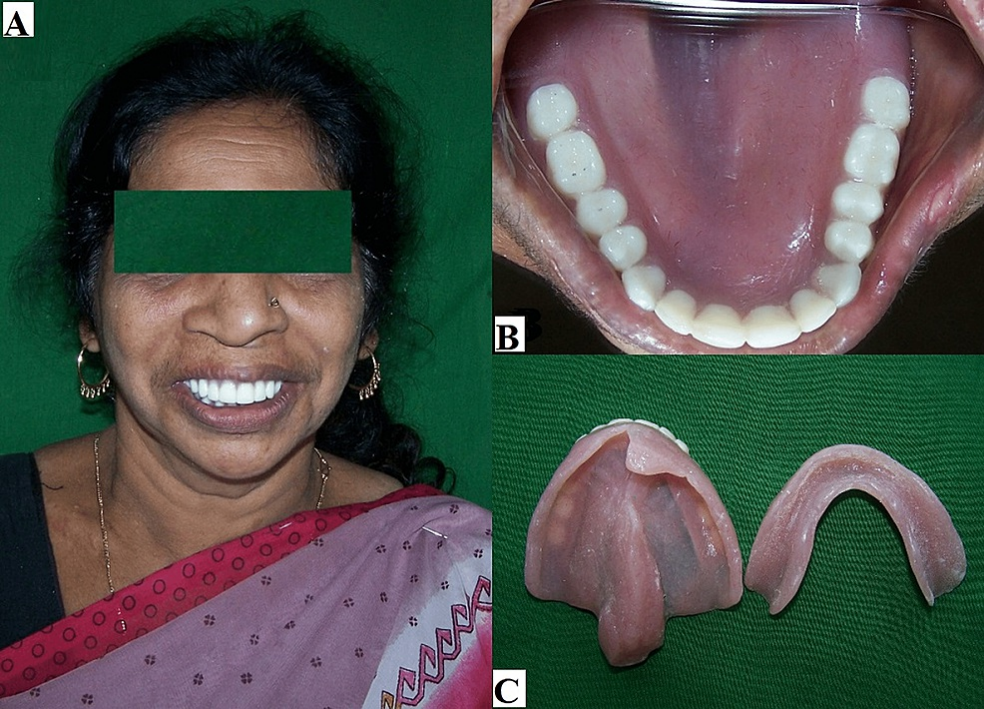
(A) Posttreatment photograph of the patient with dentures. (B) Intraoral view with dentures. (C) Patient's dentures.

The waxed dentures were invested and dewaxed using conventional techniques, according to the manufacturer’s instructions. Complete dentures were delivered to the patient (Figure 5).

All the parameters followed during the try-on were verified again during denture insertion. The patient was instructed on how to clean and maintain the complete dentures. The patient was satisfied with the aesthetics, speech, swallowing, and chewing capacity due to improved retention and stability in the new prosthesis. The patient was recalled for one week, one month, and six months to assess any difficulty while using the complete dentures.

## Discussion

The most common craniofacial congenital birth defect, with significant variation across ethnic groups and geographic regions, is the cleft lip/palate. It is classified as syndromic or nonsyndromic based on its association with other anomalies [1]. A cleft lip/palate has a multifactorial etiology that is caused by gene-gene and gene-environmental interactions.

It has huge psychological, social, medical, and financial ramifications. In developing countries, treatment is dictated by socioeconomic factors and access to medical facilities. The patient had surgical closure of the lip during early childhood. The patient could not remember exactly when the surgical closure of the lip was done. The patient’s low financial status during childhood prevented surgical closure of the palate. An interdisciplinary approach in a timely fashion works best to achieve normality in all aspects, including function and aesthetics.

Depending on the extent of the defect, patients with a complete edentulous cleft palate receive a complete denture with an obturator and pharyngeal bulb [3-8]. The prosthesis should not only improve the patient’s quality of life but also provide functional and psychological benefits. The patient was an old denture wearer and had difficulty chewing and speaking due to reduced retention and stability. A proper peripheral seal and intimate contact of the dentures with the oral mucosa provide retention for the maxillary denture. The upward extension of the obturator compensated for the lesser seal provided by the posterior palatal area.

The final weight of the maxillary denture was a little heavy but the same as her old denture. Hollow obturator is always the best option. But in this case, the obturator area had not much thickness to make it hollow. Second, the patient's straightforward request was to keep it same as her old denture. The patient could use her new denture very well due to her outstanding skill in using her old dentures. The patient's skills play an important role in the stability of dentures [9]. However, every patient needs to be uniquely handled depending on their needs.

The patient showed remarkable improvement in the speech when evaluated by speech tests like perceptual analyses, resonance frequency analyses, and acoustic analyses. The denture also prevented regurgitation of liquid and food during swallowing. During recall visits, the patient was satisfied with the dentures in terms of aesthetics, chewing, swallowing, and speech.

## Conclusions

Cleft lip and palate present a challenge during treatment and require long-term treatment planning and constant follow-up. The case report presents the successful management of an edentulous cleft palate patient with a maxillary complete denture having a palatal obturator and pharyngeal extension. The patient was satisfied with dentures in terms of aesthetics, chewing, swallowing, and speech. The prosthetic rehabilitation of a cleft palate is not only cheap and fast but also a conservative approach with fewer complications.

## References

[1] Slayton RL, Williams L, Murray J (2003). Genetic association studies of cleft lip and/or palate with hypodontia outside the cleft region. Cleft Palate Craniofac J.

[2] Hickey AJ, Salter M (2006). Prosthodontic and psychological factors in treating patients with congenital and craniofacial defects. J Prosthet Dent.

[3] Abadi B, Johnson JD (1982). The prosthodontic management of cleft palate patients. J Prosthet Dent.

[4] Montero J, Macedo C, Rodriguez M (2011). Prosthetic rehabilitation of an edentulous cleft palate using a denture with a palatal obturator: a clinical report. J Clin Exp Dent.

[5] Mazaheri M (1964). Prosthodontics in cleft palate treatment and research. J Prosthet Dent.

[6] Reisberg DJ (2000). Dental and prosthodontic care for patients with cleft or craniofacial conditions. Cleft Palate Craniofac J.

[7] Walter JD (2005). Obturators for cleft palate and other speech appliances. Dent Update.

[8] Lund TW, Wade M (1993). Use of osseointegrated implants to support a maxillary denture for a patient with repaired cleft lip and palate. Cleft Palate Craniofac J.

[9] Winkler S (1988). Essentials of Complete Denture Prosthodontics, 2nd edition, 332 pp. Littleton: PSG Publishing.

